# Association between polymorphisms in *PRNCR1* and risk of colorectal cancer in the Saudi population

**DOI:** 10.1371/journal.pone.0220931

**Published:** 2019-09-05

**Authors:** Mohammad AlMutairi, Narasimha Reddy Parine, Jilani Purusottapatnam Shaik, Sooad Aldhaian, Nahla A. Azzam, Abdulrahman M. Aljebreen, Othman Alharbi, Majid A. Almadi, Amal O. Al-Balbeesi, Mohammad Alanazi

**Affiliations:** 1 Genome Research Chair, Department of Biochemistry, College of Science King Saud University, Riyadh, Kingdom of Saudi Arabia; 2 College of Medicine, King Saud University, Riyadh, Kingdom of Saudi Arabia; 3 Division of Gastroenterology, King Khalid University Hospital, King Saud University, Riyadh, Kingdom of Saudi Arabia; 4 Department of Dermatology, King Khalid University Hospital, King Saud University, King Saud University, Riyadh, Kingdom of Saudi Arabia; King Abdulaziz University Hospital, SAUDI ARABIA

## Abstract

LncRNA Prostate cancer non-coding RNA (*PRNCR1*) is downregulated in many types of cancer. The current case-control study was performed on 144 patients with colorectal cancer and 130 matching controls. Genotyping was performed using TaqMan assays for four Single Nucleotide Polymorphisms (SNPs) in *PRNCR1*. RNAsnp Web Server was used to detect variations in the secondary structure for each SNP. The genotyping analysis for SNP rs1456315 showed increased association with colorectal cancer with the homozygous CC variant allele (OR: 2.09; χ2 = 4.95; CI: 1.08–4.02; p = 0.02), the minor allele frequency, and additive genotype, respectively (OR: 1.55; χ2 = 6.24; CI: 1.09–2.19; p = 0.01) & (OR: 1.64; χ2 = 4.04; CI: 1.01–2.67; p = 0.04). A risk association was also observed among younger age patients (≤57) and in female patients as well as in patients with tumors of the colon. For the other SNPs tested (rs16901946, rs13252298, rs1016343), no significant association was observed. The secondary structure of the rs1456315 mutant is different from that of the wild-type. Our findings suggest that the upregulation of *PRNCR1* and its variants is associated with increased risk of colorectal cancer in Saudi patients, indicating that *PRNCR1* might be a unique and valuable signature for predicting the risk of colorectal cancer in a Saudi population.

## Introduction

Colorectal cancer develops due to the accumulation of a series of epigenetic and genetic variations. Several biological pathways may drive the development of epithelial cells into colorectal adenocarcinoma. The genetic basis for colorectal cancer is depicted as a multistep model of cancer development [1]. Long noncoding RNAs are described to be key genetic regulators of crucial biological processes that play roles in cancer and other diseases. In general, lncRNAs are RNA transcripts which are longer than 200 nucleotides but do not code for any proteins [2]. Some lncRNAs, such as *Prostate cancer non-coding RNA 1* (*PRNCR1*), *HOTAIR*, and *UCA1*, are believed to be involved in cancer development. Long noncoding RNA *PRNCR1* is downregulated in many types of cancer. *PRNCR1* is a ~13 kb intron-less lncRNA which is transcribed from 8q24 [3]. Previous studies found that *PRNCR1* plays a vital role in the development of prostate cancer predisposition and it might be pivotal in prostate cancer progression by modifying androgen receptor (AR) function. Recent studies demonstrated that *PRNCR1* plays a critical role in progression of several cancers [4] [5]. Yang et al. (2013) reported that *PRNCR1* enhances the chances of prostate cancer progression by changing the AR mechanism. *PRNCR1* binds to the androgen receptor acetylated region and its relationship with DOT1L seems to be mandatory for recruitment of *PCGEM* to the DOT1L-mediated methylation of AR at the N-terminus. The collaborations of these overexpressed lncRNAs could hypothetically act as essential regulators in prostate cancer. However, the relationship between lncRNA *PRNCR1* and development of colorectal cancer was previously unknown. In recent years many reports have stated that the variants of 8q24 may contribute to a predisposition for colon cancer [6]. *PRNCR1* is situated in a sensitive region of the genomic area for CRC, yet the role of *PRNCR1* is still not yet studied in Saudi populations for any cancer. Recent findings recommended that people with *PRNCR1* variants may have a higher risk of developing cancer [7]. In the present study, we planned to evaluate the association of *PRNCR1* variants with Saudi colorectal cancer progression. To achieve this goal, we sought to use genotyping and gene expression methods to evaluate the risk variants’ role and gene expression levels in Saudi colorectal cancer patients.

## Materials and methods

### Sampling and DNA isolation

A total of 144 colon cancer blood samples were collected from at endoscopy department of King Khalid University Hospital, King Saud University from 2012 to 2017 as per the guidelines of IRB. The study protocol was reviewed and approved by the local ethic committee from KKUH (Project approval Number E12-596), and all study patients gave their written informed consent. The diagnosis and confirmation of colorectal cancer were done based on endoscopy and histopathological results. Simultaneously, 130 healthy age- and sex-matched individuals were recruited as controls. The demographic and clinical data of the patients and controls are presented in Table 1. DNA was isolated from peripheral blood samples using the Qiagen blood DNA kit.

**Table 1:** Clinical and demographic characteristics of the study subjects. Percentage of total cases are shown in parentheses.

Clinical characteristic		CRC Cases	Controls
	Number	144	130
**Gender**	Male	83 (57.64)	71 (54.62)
	Female	61 (42.36)	59 (45.38)
**Age**	< 58	66 (45.14)	76 (58.46)
	> 58	78 (54.86)	54 (41.54)
**Tumor location**	Colon	91 (63.19)	--
	Rectum	53 (36.81)	--
**Tumor stage**	I	19 (13.19)	
	II	50 (34.72)	
	III	58 (40.28)	
	IV	17 (11.81)	

### Genotyping

Based on previous reports, four SNPs, rs1016343 (C_7531214_10), rs13252298 (C_1645362_20), rs16901946 (C_33280498_20), and rs1456315 (C_7531200_20) in *PRNCR1* were selected for the present study. Genotyping was performed using TaqMan genotyping assays (Applied biosystems, USA) in colorectal cancer (n = 144) and matched peripheral blood DNA samples (n = 130) (S1 and S2 Tables). Genotyping experiments were conducted using Quant Studio 7 (Applied Biosystems, USA) as described by Alanazi et al. [8].

### Statistical analyses

A χ^2^ test was used to evaluate the demographic differences, variables, and genotypes of the PRNCR1 polymorphic variants; the Hardy-Weinberg equilibrium was tested by a χ^2^ adjustment test to compare the frequencies of the genotypes observed with the frequencies expected among the controls. Odds ratios, confidence intervals, and significance were calculated using a web tool https://ihg.gsf.de/cgi-bin/hw/hwa1.pl. Bonferroni correction was used for multiple comparison of p-value. RNAsnp Web Server (https://rth.dk/resources/rnasnp/) [9], was used to predict the secondary structure for PRNCR1 variants with each SNP.

## Results

The present case-control study comprised 144 colorectal cancer patients and 130 healthy subjects that closely matched the patients in terms of age and sex. The clinical data of the patients are presented in Table 1. The patients had a median age of 57 years (range 23–79 years) with 83 males and 61 females. A total of 91 patients had colon cancer while 53 patients had rectal cancer. All stages of CRC were included in the study: stage I (19 samples), stage II (50 samples), stage III (58 samples) and stage IV (17 samples). The healthy subjects had a median age of 57 years (range 23–79 years) with 71 males and 59 females.

All of the four SNPs, rs1016343 (C>T), rs13252298 (A>G), rs16901946 (A>G) and rs1456315 (T>C), followed Hardy Weinberg Equilibrium. Minor allele frequencies and p values of the SNPs are shown in Table 2.

**Table 2:** Primary information for *PRNCR1* polymorphisms.

Genotyped SNPs	rs1016343	rs13252298	rs16901946	rs1456315
Chromosome	8	8	8	8
Chr Pos	127081052	127082911	127088680	127091692
Base change	C>T	A>G	A>G	T>C
MAF in our controls	0.16	0.28	0.02	0.48
P value for HWE^b^ test in our controls	0.54	1.0	1.0	0.08

Out of the four SNPs only one SNP, rs1456315, showed significant risk association with colorectal cancer. Table 3 shows the obtained genotype and allele frequencies and significance of the genotype and allele distribution of the tested SNPs. The results showed that PRNCR1 SNP rs1456315 had a statistically significant risk association with Saudi CRC patients. The homozygous variant “CC” genotype showed significant association (OR: 2.09; χ2 = 4.95; CI: 1.086–4.028; p = 0.02). The minor allele “C” and additive genotype “TC+CC” also showed significant association (OR: 1.55; χ2 = 6.24; CI: 1.098–2.193; p = 0.01) & (OR: 1.64; χ2 = 4.04; CI: 1.011–2.675; p = 0.04), respectively. Only SNP rs1456315 minor allele C showed significant association even after Bonferroni correction (p = 0.04). The remaining three SNPs rs1016343 (C>T), rs13252298 (A>G), and rs16901946 (A>G) did not show any association with colorectal cancer (Table 3).

**Table 3:** Relationships of polymorphisms in *PRNCR1* gene and colorectal cancer susceptibility. Percentage of total cases are shown in parentheses.

SNP	Variant	patients Cases	Controls	OR	CI	χ^2^ Value	P- Value
rs1456315	TT	50(0.35)	61(0.47)	Ref			
	TC	57(0.40)	48(0.37)	1.449	0.848–2.476	1.84	0.17458
	CC	36(0.25)	21(0.16)	2.091	1.086–4.028	4.95	0.02617 ^ns^
	TC+CC	93(0.65)	69(0.53)	1.644	1.011–2.675	4.04	0.04455 ^ns^
	T	157(0.55)	170(0.65)	Ref			
	C	129(0.45)	90(0.35)	1.552	1.098–2.193	6.24	0.01250
rs16901946	AA	140(0.97)	128(0.98)	Ref			
	AG	4(0.03)	2(0.02)	1.829	0.329–10.153	0.49	0.48396
	GG	0	0	0.915	0.018–46.430	0.01	1.00000
	AG+GG	4(0.03)	2(0.02)	1.829	0.329–10.153	0.49	0.48396
	A	284(0.99)	258(0.99)	Ref			
	G	4(0.01)	2(0.01)	1.817	0.330–10.003	0.48	0.68981
rs13252298	AA	74(0.51)	58(0.47)	Ref			
	AG	59(0.41)	51(0.41)	0.907	0.545–1.508	0.14	0.70586
	GG	11(0.08)	14(0.11)	0.616	0.260–1.457	1.23	0.26713
	AG+GG	70(0.49)	65(0.53)	0.844	0.521–1.367	0.48	0.49031
	A	207(0.72)	167(0.68)	Ref			
	G	81(0.28)	79(0.32)	0.827	0.571–1.199	1.01	0.31588
rs1016343	CC	100(0.70)	92(0.71)	Ref			
	CT	37(0.26)	34(0.26)	1.001	0.581–1.727	0.001	0.99663
	TT	5(0.04)	3(0.02)	1.533	0.356–6.596	0.33	0.56321
	CT+CC	42(0.30)	37(0.29)	1.044	0.618–1.765	0.03	0.87133
	C	237(0.83)	218(0.84)	Ref			
	T	47(0.17)	40(0.16)	1.081	0.682–1.712	0.11	0.74055

^ns^ = not significant after Bonferroni correction

To check the association of PRNCR1 SNPs with age, gender, and tumor location of the patients, we did a further stratified analysis. To assess the association of PRNCR1 SNPs with age, cancer and control samples were stratified into two groups split on median age as ≤ 57 years, and >57 years. The genotype frequencies of both the groups are shown in Tables 4 and 5, respectively.

**Table 4:** The association between *PRNCR1* gene polymorphism and the risk of colorectal cancer in patients younger than 58 years. Percentage of total cases are shown in parentheses.

SNP	Variant	patients Cases	Controls	OR	CI	χ^2^ Value	P- Value
rs1456315	TT	20(0.30)	39(0.51)	Ref			
	TC	28(0.42)	27(0.36)	2.022	0.95–4.303	3.38	0.06603
	CC	17(0.26)	10(0.13)	3.315	1.283–8.563	6.38	0.01152*
	TC+CC	45(0.69)	37(0.49)	2.372	1.186–4.741	6.08	0.01369*
	T	68(0.52)	105(0.69)	Ref			
	C	62(0.48)	47(0.31)	2.037	1.252–3.31	8.31	0.00394*
rs16901946	AA	63(0.95)	76(1.00)	Ref			
	AG	3(0.05)	0	8.433	0.428–166.329	3.53	0.06030
	GG	0	0	1.205	0.024–61.576	nan	1.00000
	AG+GG	3(0.05)	0	8.433	0.428–166.329	3.53	0.06030
	A	129(0.98)	152(1.00)	Ref			
	G	3(0.02)	0	8.243	0.422–161.067	3.49	3.49
rs13252298	AA	33(0.50)	30(0.42)	Ref			
	AG	25(0.38)	32(0.45)	0.710	0.346–1.459	0.87	0.35092
	GG	8(0.12)	9(0.13)	0.808	0.276–2.363	0.15	0.69684
	AG+GG	33(0.50)	41(0.58)	0.732	0.373–1.436	0.83	0.36333
	A	91(0.69)	92(0.65)	Ref			
	G	41(0.31)	50(0.35)	0.829	0.501–1.373	0.53	0.46605
rs1016343	CC	51(0.78)	53(0.70)	Ref			
	CT	14(0.22)	21(0.28)	0.693	0.318–1.508	0.86	0.35393
	TT	0	1(0.02)	0.346	0.014–8.696	0.95	0.32882
	CT+CC	14(0.22)	22(0.29)	0.661	0.305–1.432	1.11	0.29260
	C	116(0.89)	127(0.85)	Ref			
	T	14(0.11)	23(0.15)	0.666	0.327–1.356	1.27	0.26070

* = significant after Bonferroni correction

**Table 5:** The association between *PRNCR1* gene polymorphism and the risk of colorectal cancer in patients older than 58 years. Percentage of total cases are shown in parentheses.

SNP	Variant	patients Cases	Controls	OR	CI	χ^2^ Value	P- Value
rs1456315	TT	30(0.38)	22(0.41)	Ref			
	TC	29(0.37)	21(0.39)	1.013	0.461–2.223	0.00	0.97490
	CC	19(0.25)	11(0.20)	1.267	0.503–3.192	0.25	0.61586
	TC+CC	48(0.62)	32(0.59)	1.100	0.541–2.235	0.07	0.79217
	T	89(0.57)	65(0.60)	Ref			
	C	67(0.43)	43(0.40)	1.138	0.691–1.874	0.26	0.61158
rs16901946	AA	77(0.99)	52(0.96)	Ref			
	AG	1(0.01)	2(0.04)	0.338	0.030–3.821	0.84	0.35868
	GG	0	0	0.677	0.013–34.677	nan	1.00000
	AG+GG	1(0.01)	2(0.04)	0.338	0.030–3.821	0.84	0.35868
	A	155(0.99)	106(0.98)	Ref			
	G	1(0.01)	2(0.02)	0.342	0.031–3.819	0.83	0.58225
rs13252298	AA	41(0.53)	28(0.54)	Ref			
	AG	34(0.44)	19(0.37)	1.222	0.584–2.559	0.28	0.59458
	GG	3(0.04)	5(0.10)	0.410	0.091–1.855	1.41	0.23563
	AG+GG	37(0.47)	24(0.46)	1.053	0.521–2.127	0.02	0.88590
	A	116(0.74)	75(0.72)	Ref			
	G	40(0.27)	29(0.28)	0.892	0.510–1.560	0.16	0.68813
rs1016343	CC	49(0.64)	39(0.72)	Ref			
	CT	23(0.30)	13(0.24)	1.408	0.633–3.133	0.71	0.40053
	TT	5(0.06)	2(0.04)	1.990	0.366–10.815	0.66	0.41819
	CT+CC	28(0.36)	15(0.28)	1.486	0.698–3.161	1.06	0.30296
	C	121(0.79)	91(0.84)	Ref			
	T	33(0.21)	17(0.16)	1.460	0.766–2.783	1.33	0.24882

SNP rs1456315, which showed significant association in the overall analysis, showed significant risk association in patients below 57 years old. The SNP rs1456315 homozygous variant “CC” genotype showed a 3.3-fold more risk association (OR: 3.31; χ2 = 6.38; CI: 1.283–8.563; p = 0.01), while “TC+CC” showed a 2.3-fold risk (OR: 2.37; χ2 = 6.08; CI: 1.186–4.741; p = 0.01), even after Bonferroni correction (p = 0.04), specifically in patients ≤ 57 years of age. The minor allele “C” showed 2-fold significant association (OR: 2.03; χ2 = 8.31; CI: 1.252–3.31; p = 0.003) in ≤ 57-year-old patients, even after Bonferroni correction (p = 0.012) (Table 4). No association was observed with > 57-year-old patients for all of the four tested SNPs (Table 5).

Further, genotype frequencies were analyzed based on gender and the data are shown in Tables 6 and 7. No statistically significant association was observed in males with *PRNCR1* SNPs (Table 6). In contrast to this, a statistically significant risk association was observed in females with the rs1456315 SNP (Table 7). The SNP rs1456315 homozygous variant “CC” frequency was 5.6 fold higher in female patients compared to those in controls and showed a significant association in female CRC patients (OR: 5.625; χ2 = 8.57; CI: 1.648–19.202; p = 0.003), even after Bonferroni correction (p = 0.012). The additive alleles of rs1456315 “TC+CC” showed significant risk association in female cases compared to controls (OR: 2.25; χ2 = 4.77; CI: 1.081–4.683; p = 0.02). The SNP rs1456315 minor allele “C” frequency was 2.3 fold higher in female patients compared to that in the matched control (OR: 2.329; χ2 = 9.36; CI: 1.348–4.026; p = 0.002), even after Bonferroni correction (p = 0.008) (Table 7).

**Table 6:** The association between *PRNCR1* gene polymorphism and the risk of colorectal cancer in male patients. Percentage of total cases are shown in parentheses.

SNP	Variant	patients Cases	Controls	OR	CI	χ^2^ Value	P- Value
rs1456315	TT	28(0.34)	28(0.39)	Ref			
	TC	33(0.40)	26(0.37)	1.269	0.609–2.644	0.41	0.52404
	CC	21(0.26)	17(0.24)	1.235	0.540–2.823	0.25	0.61616
	TC+CC	54(0.66)	43(0.61)	1.256	0.649–2.428	0.46	0.49812
	T	89(0.54)	82(0.58)	Ref			
	C	75(0.46)	60(0.42)	1.152	0.732–1.812	0.37	0.54113
rs16901946	AA	79(0.95)	70(0.99)	Ref			
	AG	4(0.05)	1(0.01)	3.544	0.387–32.464	1.42	0.23387
	GG	0	0	0.887	0.017–45.280	nan	1.00000
	AG+GG	4(0.05)	1(0.01)	3.544	0.387–32.464	1.42	0.23387
	A	162(0.98)	141(0.99)	Ref			
	G	4(0.02)	1(0.01)	3.481	0.385–31.512	1.39	0.38167
rs13252298	AA	40(0.48)	35(0.51)	Ref			
	AG	36(0.43)	26(0.38)	1.212	0.615–2.388	0.31	0.57916
	GG	7(0.08)	8(0.12)	0.766	0.252–2.326	0.22	0.63702
	AG+GG	43(0.52)	34(0.49)	1.107	0.584–2.096	0.10	0.75592
	A	116(0.70)	96(0.70)	Ref			
	G	50(0.30)	42(0.30)	0.985	0.603–1.610	0.00	0.95264
rs1016343	CC	60(0.73)	46(0.65)	Ref			
	CT	19(0.23)	23(0.32)	0.633	0.309–1.300	1.56	0.21145
	TT	3(0.04)	2(0.03)	1.150	0.184–7.169	0.02	0.88092
	CT+CC	22(0.27)	25(0.35)	0.675	0.338–1.345	1.26	0.26237
	C	139(0.85)	115(0.81)	Ref			
	T	25(0.15)	27(0.19)	0.766	0.421–1.392	0.77	0.38118

**Table 7:** The association between *PRNCR1* gene polymorphism and the risk of colorectal cancer in female patients. Percentage of total cases are shown in parentheses.

SNP	Variant	patients Cases	Controls	OR	CI	χ^2^ Value	P- Value
rs1456315	TT	22(0.36)	33(0.56)	Ref			
	TC	24(0.39)	22(0.37)	1.636	0.742–3.609	1.50	0.22115
	CC	15(0.25)	4(0.07)	5.625	1.648–19.202	8.57	0.00342*
	TC+CC	39(0.64)	26(0.44)	2.250	1.081–4.683	4.77	0.02899 ^ns^
	T	68(0.56)	88(0.70)	Ref			
	C	54(0.44)	30(0.25)	2.329	1.348–4.026	9.36	0.00222*
rs16901946	AA	61(1.00)	58(0.98)	Ref			
	AG	0	1(0.02)	0.317	0.013–7.940	1.04	0.30722
	GG	0	0	0.951	0.019–48.727	nan	1.00000
	AG+GG	0	1(0.02)	0.317	0.013–7.940	1.04	0.30722
	A	122(1.00)	117(0.99)	Ref			
	G	0	1(0.01)	0.320	0.013–7.927	1.04	0.81875
rs13252298	AA	34(0.56)	23(0.43)	Ref			
	AG	23(0.38)	25(0.46)	0.622	0.287–1.351	1.45	0.22928
	GG	4(0.07)	6(0.11)	0.451	0.114–1.777	1.34	0.24739
	AG+GG	27(0.44)	31(0.57)	0.589	0.281–1.234	1.98	0.15940
	A	91(0.75)	71(0.66)	Ref			
	G	31(0.25)	37(0.34)	0.654	0.370–1.155	2.15	0.14217
rs1016343	CC	40(0.67)	46(0.79)	Ref			
	CT	18(0.30)	11(0.19)	1.882	0.795–4.454	2.10	0.14733
	TT	2(0.03)	1(0.02)	2.300	0.201–26.324	0.47	0.49183
	CT+CC	20(0.33)	12(0.21)	1.917	0.834–4.403	2.39	0.12247
	C	98(0.82)	103(0.89)	Ref			
	T	22(0.18)	13(0.11)	1.779	0.849–3.725	2.37	0.12356

^ns^ = not significant after Bonferroni correction,

* = significant after correction

The association of PRNCR1 SNPs with CRC was also analyzed by stratifying the tumor samples based on tumor location. The patients were divided into those with tumors located in the colon region and those with tumors in the rectal region. Interestingly, rs1456315 showed statistically significant association in the colon while in rectal cancer no significant association was observed in CRC patients (Tables 8 and 9).

**Table 8:** Genotype frequencies of *PRNCR1* gene polymorphism in colorectal tumors located in the colon area. Percentage of total cases are shown in parentheses.

SNP	Variant	patients Cases	Controls	OR	CI	χ^2^ Value	P- Value
rs1456315	TT	30(0.33)	61(0.47)	Ref			
	TC	38(0.42)	48(0.37)	1.610	0.875–2.963	2.35	0.12510
	CC	23(0.25)	21(0.16)	2.227	1.067–4.647	4.64	0.03131 ^ns^
	TC+CC	61(0.67)	69(0.53)	1.798	1.030–3.136	4.30	0.03801 ^ns^
	T	98(0.54)	170(0.65)	Ref			
	C	84(0.46)	90(0.35)	1.619	1.099–2.385	5.97	0.01454*
rs16901946	AA	90(0.99)	128(0.98)	Ref			
	AG	1(0.01)	2(0.02)	0.711	0.064–7.962	0.08	0.78107
	GG	0	0	1.420	0.028–72.220	0.001	1.00000
	AG+GG	1(0.01)	2(0.02)	0.711	0.064–7.962	0.08	0.78107
	A	181(0.99)	258(0.99)	Ref			
	G	1(0.01)	2(0.01)	0.713	0.064–7.919	0.08	1.09652
rs13252298	AA	47(0.52)	58(0.47)	Ref			
	AG	38(0.42)	51(0.41)	0.919	0.520–1.625	0.08	0.77266
	GG	6(0.07)	14(0.11)	0.529	0.189–1.483	1.50	0.22082
	AG+GG	44(0.48)	65(0.53)	0.835	0.486–1.437	0.42	0.51561
	A	132(0.73)	167(0.68)	Ref			
	G	50(0.27)	79(0.32)	0.801	0.525–1.220	1.07	0.30090
rs1016343	CC	63(0.70)	92(0.71)	Ref			
	CT	23(0.26)	34(0.26)	0.988	0.532–1.834	0.00	0.96914
	TT	4(0.04)	3(0.02)	1.947	0.421–9.000	0.75	0.38597
	CT+CC	27(0.30)	37(0.29)	1.066	0.590–1.924	0.04	0.83290
	C	149(0.83)	218(0.84)	Ref			
	T	31(0.17)	40(0.16)	1.134	0.679–1.894	0.23	0.63116

^ns^ = not significant after Bonferroni correction,

* = significant after correction

**Table 9:** Genotype frequencies of *PRNCR1* gene polymorphism in colorectal cancer tumors located in the rectal area. Percentage of total cases are shown in parentheses.

SNP	Variant	patients Cases	Controls	OR	CI	χ^2^ Value	P- Value
rs1456315	TT	20(0.38)	61(0.47)	Ref			
	TC	19(0.37)	48(0.37)	1.207	0.580–2.513	0.25	0.61424
	CC	13(0.25)	21(0.16)	1.888	0.802–4.446	2.15	0.14285
	TC+CC	32(0.62)	69(0.53)	1.414	0.734–2.727	1.08	0.29943
	T	59(0.57)	170(0.65)	Ref			
	C	45(0.43)	90(0.35)	1.441	0.905–2.292	2.38	0.12256
rs16901946	AA	50(0.94)	128(0.98)	Ref			
	AG	3(0.06)	2(0.02)	3.840	0.623–23.672	2.41	0.12079
	GG	0	0	2.545	0.050–129.979	0.001	1.00000
	AG+GG	3(0.06)	2(0.02)	3.840	0.623–23.672	2.41	0.12079
	A	103(0.97)	258(0.99)	Ref			
	G	3(0.03)	2(0.01)	3.757	0.619–22.815	2.37	0.25226
rs13252298	AA	27(0.51)	58(0.47)	Ref			
	AG	21(0.40)	51(0.41)	0.885	0.447–1.752	0.12	0.72478
	GG	5(0.09)	14(0.11)	0.767	0.251–2.348	0.22	0.64176
	AG+GG	26(0.49)	65(0.53)	0.859	0.451–1.637	0.21	0.64447
	A	75(0.71)	167(0.68)	Ref			
	G	31(0.29)	79(0.32)	0.874	0.532–1.436	0.28	0.59427
rs1016343	CC	37(0.71)	92(0.71)	Ref			
	CT	14(0.27)	34(0.26)	1.024	0.493–2.125	0.00	0.94955
	TT	1(0.02)	3(0.02)	0.829	0.084–8.227	0.03	0.87245
	CT+CC	15(0.29)	37(0.29)	1.008	0.495–2.052	0.00	0.98240
	C	88(0.85)	218(0.84)	Ref			
	T	16(0.15)	40(0.16)	0.991	0.528–1.861	0.00	0.97735

Patients with colon cancer showed significantly higher risk (2.2 fold) with SNP rs1456315 homozygous variant genotype “CC” when compared to healthy individuals (OR: 2.227; χ2 = 4.64; CI: 1.067–4.647; p = 0.03). The additive alleles “TC+CC” genotype showed significant association in CRC patients compared to healthy individuals (OR: 1.798; χ2 = 4.30; CI: 1.030–3.136; p = 0.03). The minor allele “C” frequency was also showed significantly higher association in patients when compared to healthy controls (OR: 1.619; χ2 = 5.97; CI: 2.385–1.099; p = 0.01), even after Bonferroni correction (p = 0.04) (Table 8). However, the genotype association was not observed for rectal CRC patients with any of the PRNCR1 SNPs (Table 9).

**Fig 1:**
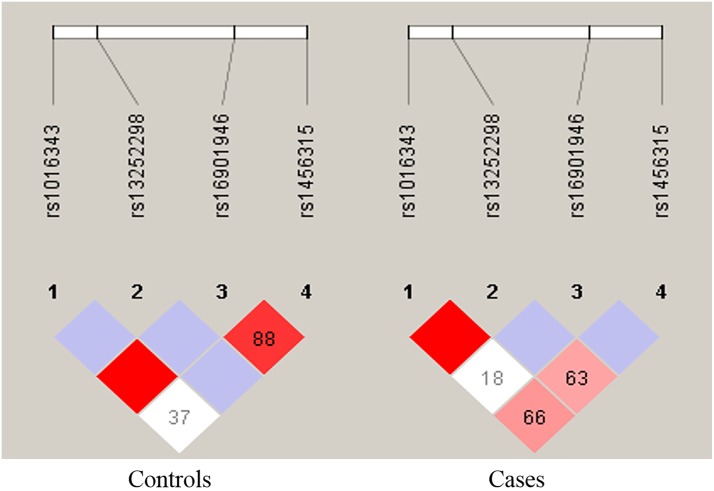
Linkage disequilibrium association among the four SNPs in colon cancer and controls. The thick red color indicates the higher r^2^ value.

Linkage disequilibrium analysis showed that there was very low association among the analyzed SNPs in controls. The r^2^ values are slightly different in colon cancer cases compared to controls (Fig 1). LDproxy analysis for rs1456315 showed a close association with rs13254738 (r^2^ = 0.66). LDproxy analysis of rs16901946 showed a close association with four PRNCR1 SNPs rs12682421 (r^2^ = 1), rs77236771 (r^2^ = 0.89), rs139756632 (r^2^ = 0.86) and rs75414904 (r^2^ = 0.86). SNP rs1016343 Showed close association with rs7837848 (r^2^ = 0.88).

**Fig 2:**
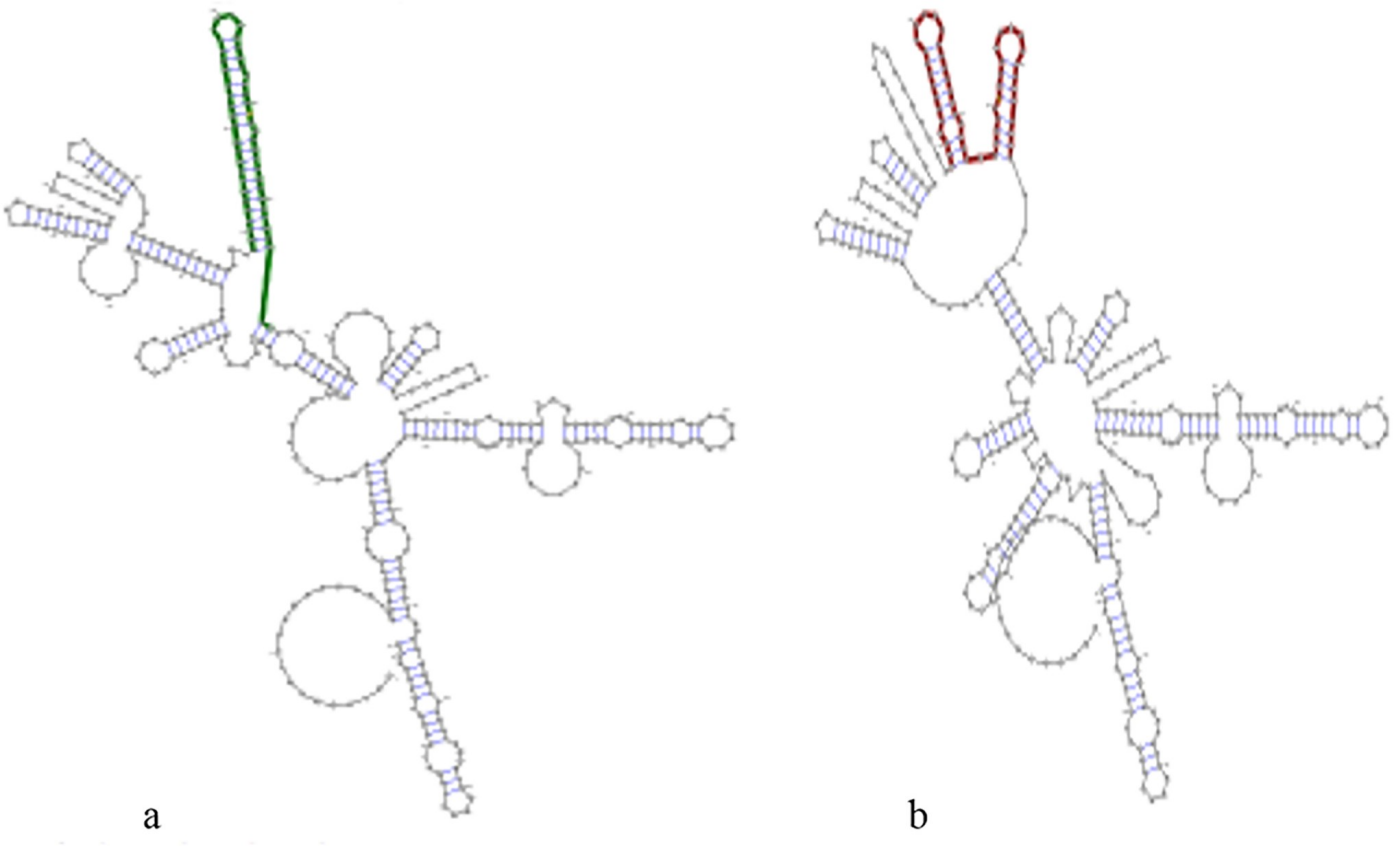
Secondary structure for *PRNCR1* rs1456315 and base pair probabilities. a) Wild-type b) Mutant.

The RNAsnp prediction showed that rs1456315 changed the RNA secondary structure of PRNCR1 (Fig 2). The RNAsnp predicted that the rs1456315 T-C allele substitution resulted in a minimum free energy (MFE) of (-85.70)–(-85.10) kcal/mol. The base pair probabilities of rs14563153 wild type (T allele) and variant (C allele) were different.

## Discussion

To the best of our knowledge, no study investigating the impact of *PRNCR1* variants on colorectal cancer association in a Saudi population has been conducted to date. All of the *PRNCR1* polymorphisms are located in the exon region [10]. Among the four SNPs included in our study, in overall case control analysis and stratified analysis, only the rs1456315 (C/T) SNP was associated with cancer risk. In colorectal cancer patients, there was a significant increase in the C/C genotype compared to controls. The remaining three SNPs, rs16901946 (A/G), rs13252298 (A/G), and rs1016343 (C/T) did not show any association. Stratified analysis of our study showed that the association between CRC and rs1456315 was substantial in a subgroup of females while for males it is not significant. Genetic polymorphism of rs1456315 affecting CRC varies between genders, which was consistent with a recent report by Wang et al. [11]. This suggested that gender-related differences are observed in lncRNA *PRNCR1*. In addition, we also found that the association between CRC and rs1456315 was significant in the female population, which indicates that the role of rs1456315 in CRC susceptibility was different in the diverse population. In stratification analysis, we observed that SNP rs1456315 was significantly associated with the tumor location of CRC. We observed that SNP rs1456315 showed significant risk with colon cancer in a Saudi population. SNP rs1456315 was associated with an increased risk of CRC, especially for younger individuals. Previous studies reported that rs1456315 was associated with the association of colorectal cancer, gastric cancer and prostate cancer [7, 12, 13]. In our study, we observed that SNP rs1456315 is significantly associated with CRC risk; this finding is in consistent with the findings of Chung et al. [14]. Chung et al. observed that *PRNCR1* has a significant role in the prostate by regulating AR activity [14]. Several other studies supported that AR also participated in the pathological development of CRC through that TGFβ pathway [15] [16].

In the present study, we observed that there is no significant association of *PRNCR1* rs16901946 genotypes in colorectal cancer patients. Our results are inconsistent with Li et al. who reported that there was no association of rs16901946 with colorectal cancer [7] and gastric cancer [12]. However, recent meta-analysis performed by Chu et al. [13] reported that the rs16901946 variant of *PRNCR1* was found to increase the risk of cancer significantly. However, He et al. [17] reported that the *PRNCR1* rs16901946 polymorphisms are significantly associated with the increased association of prostate cancer in the Eastern China population. A meta-analysis study by Huang et al. [18] reported that the rs16901946 G/A polymorphism was associated with an increased overall association to cancer.

A meta-analysis study showed among the *PRNCR1* rs13252298 genotypes, the G/A homozygous variant is associated with cancer [18]. Li et al. reported that the rs13252298 of *PRNCR1* is associated with significantly less risk of CRC [7]. A recent study in Iran reported that the *PRNCR1* rs13252298 polymorphisms are significantly associated with the increased association of PCa in an Iranian population [19]. This is in contrast with our present study. There is no significant association with the SNP rs13252298 for the Saudi population.

Huang et al. reported that the SNP of *PRNCR1* rs1016343 T/C polymorphism was associated with an increased overall incidence of cancer [18]. Chu et al. reported that the rs1016343 increased the risk of cancer in the dominant model and additive model with a 24% increased risk of cancer [13]. In the meta-analysis study, they found that PRNCR1 rs1016343 and rs16901946 polymorphisms were contributing to cancer risk [10], while for the Saudi population in this study there is no significant association of rs1016343 with colorectal cancer.

Recent studies demonstrated that *PRNCR1* plays a vital role in cancer progression. One of the studies by Yang et al. [20] described that *PRNCR1* affects and alters the androgen receptor mechanism and thus increases the risk of prostate cancer development. *PRNCR1* binds to the androgen receptor acetylated region and its relationship with DOT1L seems to be mandatory for recruitment of *PCGEM* to the DOT1L-mediated methylation of AR at the N-terminus [20]. Li et al. [7] found that a critical region of 8q24 might indicate predisposition to CRC. Yang et al. [3] also specified that overexpression of *PRNCR1* was evidently interconnected with tumor stage and size. Furthermore, it has been found that patients with an rs1456315G polymorphism have a tumor of much larger size. It may be due to the effect of the polymorphisms on the secondary structure of *PRNCR1* mRNA and thus changing its stability. Further, they also reported that the silencing of *PRNCR1* inhibited the cell cycle at the G0/G1 stage demonstrating that *PRNCR1* stimulates the propagation of colon cancer from epithelial cells, and ultimately might increase the tumor size in patients. Li et al. [7] reported that 5 SNPs in *PRNCR1*: rs1456315G, rs7007694C, rs13252298, rs1456315, and rs16901946G, showed a significant association with colorectal cancer progression. Furthermore, an increasing number of GWAS studies reported a strong association of a CpG site at Chr8: 128167809 in *PRNCR1* and CRC predisposition. The *PRNCR1* SNP rs1456315G and this CpG site have been reported to have strong association with each other.

The *PRNCR1* catalytic subunit has been widely linked to cancer development. *PRNCR1* was reported to be frequently mutated in many types of cancers, such as esophageal cancer, colorectal cancer, and breast cancer. Recently, *PRNCR1* genomic variants were identified to be associated with increased cancer risk. Park et al. reported that the *PRNCR1* rs1456315G and rs6983267 SNPs were significantly associated with melanoma patient survival. Wang et al. reported that PIK3CA rs2699887 showed notable associations with the survival of endometrial cancer patients. However, the association between rs6983267 and colorectal cancer risk as well as patient survival remains elusive.

The RNAsnp prediction showed that rs1456315 changed the RNA secondary structure of PRNCR1 (Fig 2). Therefore, we speculate that rs1456315 could be a regulatory SNP, which regulates the expression of PRNCR1 and contributes to the genetic susceptibility of colorectal cancer. The RNAsnp prediction for rs16901946 showed that there is no change in the RNA structures of PRNCR1, while the base pair probabilities of rs16901946 are slightly different. The RNAsnp prediction showed that rs13252298 and rs1016343 did not changed in the folding structures of *PRNCR1*, and also the base pair probabilities are the same.

The present study has some strengths and limitations. Mainly, the genetic association analysis was performed in a small cohort, and these results must be validated in larger cohorts and other populations. We enrolled native Saudi colorectal cancer patients and controls in the present study. This is the first study to report PRNCR1 variant association with cancer in a Saudi population. In conclusion, in the present study we confirmed the association of rs1456315 with colorectal cancer risk in a Saudi population.

## Supporting information

S1 Table Control data. (PDF)

S2 Table Cases data. (PDF)
