# Supplementary material for: Association between polymorphisms in PRNCR1 and risk of colorectal cancer in the Saudi population
Source: PLoS One. 2019 Sep 5;14(9):e0220931. doi: 10.1371/journal.pone.0220931 (PMC6728072; doi:10.1371/journal.pone.0220931)
Supplement: S1 Table — (PDF) [file pone.0220931.s001.pdf]

rs1456315	rs13252298	rs16901946	rrs1016343
1	2	1	1
1	1	1	1
1	1	1	1
1	3	1	1
2	1	1	1
3	1	1	1
1	2	1	1
1	2	1	1
2	1	1	1
2	1	1	1
2	1	1	2
2	2	1	2
2	2	1	1
2	2	1	2
1	2	1	1
1	2	1	1
1	1	1	3
1	2	1	1
1	1	1	1
1	3	1	1
1	1	1	2
1	3	1	1
2	1	1	2
2	2	1	1
2	2	1	2
1	2	1	1
2	1	1	1
3	2	1	1
2	1	2	1
3	1	1	2
2	2	1	2
3	1	1	2
2	2	1	1
3	1	1	3
3	2	1	2
2	2	1	2
2	1	1	2
1	3	1	1
2	1	1	2
3	1	1	1
2	2	1	2
2	2	1	2
2	1	1	1
3	1	1	1
2	2	1	2
1	3	1	1

1= Homozygous
2= Heterozygous
3=Homozygous mutant

1	3	1	1
2	2	1	2
1	2	1	1
3	1	1	2
1	3	1	1
2	1	1	2
1	2	1	2
2	2	1	1
1	3	1	1
3	0	1	2
3	1	1	2
1	2	1	1
2	0	1	1
2	2	1	1
1	2	1	1
2	1	2	1
2	1	1	1
3	1	1	1
2	1	1	1
2	1	1	2
2	2	1	1
2	0	1	1
3	1	1	1
1	3	1	1
1	3	1	1
2	0	1	2
1	2	1	1
1	1	1	2
2	1	1	1
2	2	1	1
1	1	1	1
1	1	1	1
1	1	1	1
1	1	1	1
1	1	1	1
1	1	1	1
1	2	1	1
1	2	1	1
1	1	1	1
1	2	1	1
2	1	1	1
2	1	1	0
1	2	1	1
1	2	1	2
2	2	1	1
1	2	1	2
1	1	1	2

2	1	1	2
1	2	1	1
1	3	1	1
1	1	1	1
2	2	1	2
2	1	1	1
3	0	1	1
3	1	1	1
1	2	1	1
3	0	1	1
3	1	1	1
2	2	1	1
3	1	1	2
2	2	1	1
1	1	1	1
1	2	1	2
1	1	1	1
2	1	1	1
2	2	1	1
2	2	1	1
1	1	1	2
1	2	1	1
1	2	1	1
1	2	1	1
2	0	1	2
3	1	1	1
1	3	1	1
1	3	1	1
1	2	1	1
1	1	1	1
1	1	1	1
2	2	1	1
1	1	1	3
1	2	1	1
1	3	1	1
3	1	1	1
3	1	1	1
