# Supplementary material for: Association between polymorphisms in PRNCR1 and risk of colorectal cancer in the Saudi population
Source: PLoS One. 2019 Sep 5;14(9):e0220931. doi: 10.1371/journal.pone.0220931 (PMC6728072; doi:10.1371/journal.pone.0220931)
Supplement: S2 Table — (PDF) [file pone.0220931.s002.pdf]

sampleno	rs1456315	rs1690194	rs1325229	rs1016343	
1.00	1.00	1.00	2.00	1.00	
2.00	3.00	1.00	1.00	2.00	
3.00	1.00	1.00	1.00	1.00	
4.00	1.00	1.00	1.00	1.00	
5.00	2.00	1.00	1.00	1.00	1= Homozygous
6.00	2.00	1.00	1.00	2.00	2= Heterozygous
7.00	2.00	1.00	1.00	1.00	3=Homozygous mutant
8.00	2.00	1.00	2.00	1.00	
9.00	1.00	1.00	1.00	1.00	
10.00	3.00	1.00	1.00	2.00	
11.00	2.00	1.00	2.00	2.00	
12.00	3.00	1.00	1.00	0.00	
13.00	3.00	1.00	1.00	1.00	
14.00	2.00	1.00	3.00	1.00	
15.00	3.00	1.00	1.00	3.00	
16.00	3.00	1.00	1.00	1.00	
17.00	2.00	1.00	2.00	1.00	
18.00	2.00	1.00	1.00	1.00	
19.00	1.00	1.00	3.00	1.00	
20.00	1.00	1.00	1.00	1.00	
21.00	3.00	1.00	1.00	3.00	
22.00	1.00	1.00	3.00	1.00	
23.00	1.00	1.00	1.00	1.00	
24.00	1.00	1.00	2.00	1.00	
25.00	2.00	1.00	1.00	1.00	
26.00	3.00	1.00	2.00	1.00	
27.00	2.00	1.00	2.00	1.00	
28.00	3.00	2.00	2.00	1.00	
29.00	2.00	1.00	3.00	1.00	
30.00	0.00	1.00	1.00	2.00	
31.00	2.00	1.00	2.00	1.00	
32.00	3.00	1.00	1.00	2.00	
33.00	2.00	1.00	2.00	2.00	
34.00	1.00	1.00	2.00	1.00	
35.00	2.00	1.00	2.00	1.00	
36.00	3.00	1.00	1.00	2.00	
37.00	3.00	1.00	1.00	2.00	
38.00	3.00	1.00	1.00	1.00	
39.00	1.00	1.00	2.00	1.00	
40.00	2.00	1.00	1.00	1.00	
41.00	2.00	1.00	1.00	1.00	
42.00	2.00	2.00	1.00	2.00	
43.00	2.00	1.00	1.00	2.00	
44.00	3.00	1.00	1.00	1.00	
45.00	2.00	1.00	2.00	1.00	
46.00	3.00	1.00	2.00	2.00	

47.00	2.00	1.00	2.00	1.00
48.00	3.00	1.00	1.00	0.00
49.00	1.00	1.00	3.00	1.00
50.00	3.00	1.00	1.00	3.00
51.00	3.00	1.00	1.00	1.00
52.00	2.00	1.00	3.00	1.00
53.00	2.00	1.00	2.00	2.00
54.00	2.00	1.00	1.00	1.00
55.00	2.00	1.00	2.00	1.00
56.00	2.00	1.00	1.00	1.00
57.00	3.00	1.00	1.00	1.00
58.00	3.00	1.00	1.00	1.00
59.00	2.00	1.00	2.00	2.00
60.00	2.00	1.00	2.00	1.00
61.00	2.00	1.00	2.00	1.00
62.00	2.00	1.00	1.00	3.00
63.00	3.00	1.00	1.00	2.00
64.00	2.00	1.00	1.00	2.00
65.00	2.00	1.00	2.00	1.00
66.00	2.00	1.00	1.00	1.00
67.00	1.00	1.00	1.00	1.00
68.00	2.00	1.00	2.00	2.00
69.00	3.00	1.00	1.00	2.00
70.00	2.00	1.00	2.00	1.00
71.00	1.00	1.00	2.00	1.00
72.00	3.00	1.00	1.00	1.00
73.00	2.00	1.00	2.00	2.00
74.00	3.00	1.00	1.00	2.00
76.00	2.00	1.00	3.00	1.00
77.00	3.00	1.00	1.00	2.00
78.00	2.00	1.00	2.00	2.00
79.00	3.00	1.00	1.00	2.00
80.00	1.00	1.00	1.00	1.00
81.00	2.00	1.00	2.00	1.00
82.00	2.00	1.00	1.00	1.00
83.00	2.00	1.00	1.00	1.00
84.00	1.00	1.00	2.00	1.00
85.00	1.00	1.00	1.00	1.00
86.00	1.00	1.00	1.00	2.00
87.00	1.00	1.00	2.00	1.00
88.00	1.00	1.00	1.00	1.00
89.00	2.00	1.00	1.00	1.00
90.00	3.00	1.00	1.00	2.00
91.00	3.00	2.00	1.00	1.00
92.00	2.00	1.00	2.00	1.00
93.00	2.00	1.00	2.00	1.00
94.00	1.00	1.00	2.00	1.00

95.00	3.00	1.00	1.00	1.00
96.00	2.00	1.00	2.00	2.00
97.00	2.00	1.00	2.00	1.00
497.00	3.00	1.00	1.00	3.00
498.00	1.00	1.00	2.00	1.00
499.00	1.00	1.00	2.00	1.00
500.00	1.00	1.00	2.00	1.00
501.00	2.00	1.00	1.00	1.00
502.00	3.00	1.00	2.00	2.00
503.00	1.00	1.00	2.00	1.00
504.00	1.00	1.00	1.00	2.00
505.00	2.00	1.00	1.00	1.00
506.00	1.00	1.00	1.00	1.00
507.00	1.00	1.00	2.00	2.00
508.00	1.00	1.00	2.00	1.00
509.00	1.00	1.00	2.00	1.00
510.00	1.00	1.00	3.00	1.00
511.00	1.00	1.00	2.00	1.00
512.00	1.00	1.00	2.00	1.00
513.00	2.00	1.00	2.00	1.00
514.00	1.00	1.00	3.00	1.00
515.00	1.00	1.00	1.00	1.00
516.00	3.00	1.00	2.00	1.00
517.00	2.00	1.00	1.00	1.00
524.00	1.00	1.00	2.00	1.00
525.00	1.00	1.00	2.00	1.00
526.00	3.00	2.00	1.00	1.00
527.00	1.00	1.00	2.00	1.00
528.00	2.00	1.00	1.00	2.00
529.00	1.00	1.00	1.00	1.00
530.00	2.00	1.00	1.00	1.00
531.00	3.00	1.00	1.00	2.00
532.00	2.00	1.00	1.00	2.00
533.00	1.00	1.00	2.00	1.00
534.00	1.00	1.00	2.00	1.00
535.00	2.00	1.00	2.00	1.00
536.00	1.00	1.00	2.00	1.00
537.00	1.00	1.00	2.00	1.00
538.00	1.00	1.00	2.00	1.00
539.00	1.00	1.00	2.00	1.00
540.00	2.00	1.00	1.00	2.00
541.00	2.00	1.00	1.00	2.00
542.00	1.00	1.00	1.00	1.00
543.00	2.00	1.00	1.00	1.00
544.00	2.00	1.00	3.00	1.00
545.00	3.00	1.00	1.00	2.00
546.00	1.00	1.00	3.00	1.00

547.00	1.00	1.00	2.00	2.00
548.00	1.00	1.00	1.00	2.00
549.00	1.00	1.00	2.00	1.00
550.00	3.00	1.00	1.00	1.00
